# Chromosome-scale genome assembly and annotation of *Paspalum notatum* Flüggé var. *saurae*

**DOI:** 10.1038/s41597-024-03731-0

**Published:** 2024-08-16

**Authors:** Juan Manuel Vega, Maricel Podio, Julie Orjuela, Lorena A. Siena, Silvina C. Pessino, Marie Christine Combes, Cedric Mariac, Emidio Albertini, Fulvio Pupilli, Juan Pablo A. Ortiz, Olivier Leblanc

**Affiliations:** 1 Laboratorio de Biología Molecular, Instituto de Investigaciones en Ciencias Agrarias de Rosario (IICAR) CONICET-UNR, Facultad de Ciencias Agrarias, Campo Experimental Villarino, Universidad Nacional de Rosario, Zavalla (S2125ZAA), Santa Fe, Argentina; 2 DIADE, Univ. Montpellier, CIRAD, IRD, Montpellier, France; 3 Department of Agricultural, Food and Environmental Science, University of Perugia, 06121 Perugia, Italy; 4 Institute of Biosciences and Bioresources (IBBR), National Research Council (CNR), 06128 Perugia, Italy

**Keywords:** Genomics, Plant breeding

## Abstract

*Paspalum notatum* Flüggé is an economically important subtropical fodder grass that is widely used in the Americas. Here, we report a new chromosome-scale genome assembly and annotation of a diploid biotype collected in the center of origin of the species. Using Oxford Nanopore long reads, we generated a 557.81 Mb genome assembly (N50 = 56.1 Mb) with high gene completeness (BUSCO = 98.73%). Genome annotation identified 320 Mb (57.86%) of repetitive elements and 45,074 gene models, of which 36,079 have a high level of confidence. Further characterisation included the identification of 59 miRNA precursors together with their putative targets. The present work provides a comprehensive genomic resource for *P. notatum* improvement and a reference frame for functional and evolutionary research within the genus.

## Background & Summary

**Fig. 1:**
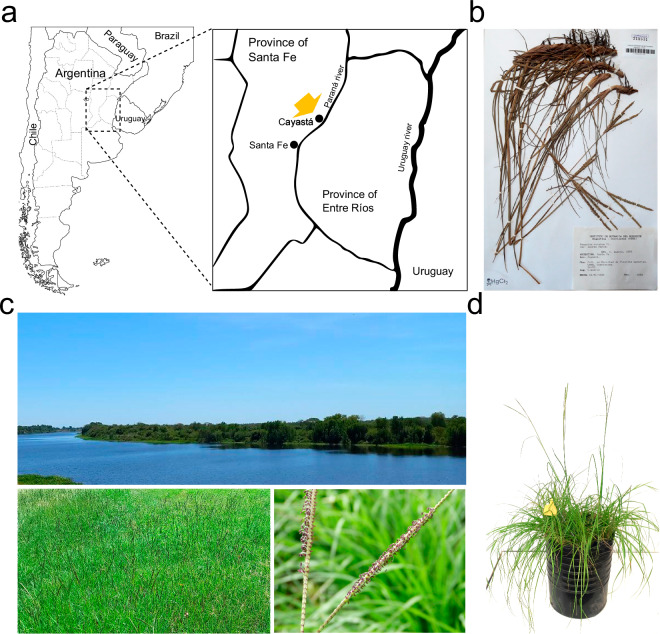
General view of the proposed center of diversity of *P. notatum*. (**a**) Map of the area of natural distribution of the diploid cytotype in the provinces of Santa Fe and Entre Ríos, Argentina. The arrow indicates the location of the city of Cayastá (31° 12′ 0″ S; 60° 10′ 0″ W), close to the sampling site of the #R1 plant. (**b**) Photograph of the herbarium voucher of diploid bahiagrass collected by Prof. Camilo Quarin in 1992 kept at the Carmen L. Cristobal herbarium at the Instituto de Botánica del Nordeste (IBONE), CONICET-UNNE, Corrientes, Argentina. (**c**) Photograph of the banks of the Paraná River in the Cayastá area (top), where natural bahiagrass populations were found (bottom left), and a close-up of a bahiagrass inflorescence at anthesis (bottom right). (**d**) Duplicate of the *P. notatum* var. *saurae* #R1 plant used for genome sequencing.

*Paspalum notatum* Flüggé (bahiagrass) is a subtropical grass native to South America that is widespread on lightly textured soils in warm, humid regions of the Western Hemisphere and extensively used as a pasture and ground cover^1,2^. The species forms a multiploid complex in which the diploid (2n = 2x = 20) plants are self-sterile and sexual, while the polyploids (3x = 30, 4x = 40, 5x = 50) are pseudogamous aposporous apomicts, *i.e*. they form seeds containing maternal embryos^3,4^. The diploid form, var. *saurae*, also known as Pensacola bahiagrass, occurs naturally in a restricted geographical area of Argentina stretching between the western and eastern banks of the Uruguay and Paraná rivers, respectively^2^. It owes its name to the fact that it was inadvertently introduced in the Pensacola area of Florida before 1926 and subsequently naturalized as a warm-season perennial pasture throughout the coastal plain and Gulf Coast regions of the United States^5^. Today, it is one of the most important grasses for pastures and lawns in the southeastern United States^6^. The search for the origin of Pensacola bahiagrass led the agricultural scientist Glenn W. Burton to travel through Brazil, Uruguay, and Argentina, where he eventually found highly diverse populations in a small area of the province of Santa Fe, on the banks of the Paraná River and the island of Berduc, near the city of Cayastá^5^ (Fig. 1a,b). Since cytogenetic studies indicate that polyploid *P. notatum* races (var. *notatum*) are autotetraploid and share homologous chromosomes with the *saurae* plants^7,8^, this region was then considered to be the center of origin of the species^2,5^.

Because *P. notatum* establishes well in poor-quality sandy soils and tolerates drought, sporadic flooding, and continuous grazing, the species has been selected and improved by classical and molecular methods for almost 80 years, with about 20 cultivars released to date^9^. While the diploid sexual races could be crossed to generate improved hybrids, tetraploid cultivars were traditionally obtained through ecotype selection due to their apomictic mode of reproduction^9^. However, the experimental production of tetraploid sexual individuals by doubling the chromosomes of diploids and the creation of synthetic sexual tetraploid populations have increased the variability for breeding programs through crosses with natural apomictic pollen donors^9–11^.

*P. notatum* ecotypes have relatively small genomes, with 1 C values ranging from 0.55 to 0.60 pg^12^. Recent studies have provided a wealth of information on the species’ genetic, transcriptomic, and genomic data^11^ and have set up strategies for the functional characterization of agronomically important genes using genetic transformation and gene editing^13–15^. Available resources include leaf and flower transcriptomes of sexual and apomictic genotypes^16–18^, a catalog of small RNAs present during the sexual and apomictic reproductive development^19^, and a chromosome-scale *de novo* genome assembly (514 Mb) of the species^20^. However, information on gene content annotation and miRNA genes is not yet available.

Long-read sequencing technologies have proven to be extremely effective in improving the quality of assembly in complex genomes, with high levels of heterozygosity, polyploidy, and repetitive elements^21–23^, particularly for non-model species and orphan crops^24–28^. Here, we report a chromosome-level genome assembly and annotation of a natural diploid *P. notatum* biotype (#R1) collected at the species center of diversity using Oxford Nanopore Technology (ONT). The plant #R1 reproduces sexually but occasionally produces aposporous embryo sacs, which is the first step of apomictic reproduction^29^. Further extensive genomic characterization using Illumina short reads, together with the existing and newly generated transcriptomes, makes the #R1 genome assembly and annotation a valuable resource for providing new insights into the gene content and genome evolution, and for elucidating the developmental genetics of agronomically valuable traits.

## Methods

### Sample collection

The #R1 plant is a diploid individual collected in a natural population established near the city of Cayastá, Santa Fe Province, Argentina^29^ (Fig. 1a,c), which belongs to the living germplasm collection of *Paspalum* spp. of the Instituto de Botánica del Nordeste (IBONE), CONICET-UNNE, Corrientes, Argentina (voucher CTES0553130; Herbarium Carmen L. Cristobal) (Fig. 1b). Several duplicates generated by vegetative propagation through rhizomes are also maintained at the Instituto de Investigaciones en Ciencias Agrarias de Rosario (IICAR), CONICET-UNR, Rosario, Argentina, and at the French National Research Institute for Sustainable development (IRD), Montpellier, France (Fig. 1d). For ONT sequencing, we used ~5 gr of fresh leaf tissue to extract high molecular weight genomic DNA (HMW gDNA) from nuclei isolation and performed quality control, both according to Mariac *et al*.^30^. We also extracted total RNA for cDNA synthesis and ONT sequencing from flowers of #R1 immature inflorescences collected before anthesis using a method adapted from Azevedo *et al*.^31^. Briefly, the plant material was ground in liquid nitrogen, mixed with the extraction buffer, incubated for 15 min at room temperature, and finally extracted using chloroform-isoamyl alcohol. We preserved RNA integrity by avoiding vortexing and keeping samples on ice throughout the extraction process. The genomic DNA used for preparing Illumina sequencing libraries was extracted from ~3 gr of fresh leaf tissue using a CTAB (cetyltrimethylammonium bromide) method^32^ and qualified for concentration and purity using a NanoDrop 2000 (Thermo Scientific, USA).

### DNA sequencing

#### Nanopore sequencing

DNA libraries of the #R1 genotype were prepared from non-fragmented HMW gDNA using the ligation sequencing Kit 1D SQK-LSK109 (Oxford Nanopore Technology). ONT sequencing was carried out using either a MinION MK1b (Oxford Nanopore Technology) at IRD or a PromethION (Oxford Nanopore Technology, UK) at Novogene (Cambridge, UK) employing R9.4.1 Spot-On Flow Cells (Oxford Nanopore Technology). ONT sequencing FAST5 files were base-called using GUPPY v6.0.6 software and the dna_r9.4.1_450bps_hac_prom.cfg model. The quality control of raw reads in FASTQ format was conducted using NanoPlot v1.31.0 software^33^.

#### Illumina sequencing

Illumina sequencing was carried out at the Instituto de Agrobiotecnología de Rosario (INDEAR; Rosario, Argentina). Sequencing libraries were prepared from 50 ng of genomic DNA using the Nextera DNA Library Prep Kit (Illumina, Inc., San Diego, CA, USA) according to the manufacturer’s instructions and sequenced using a 2 × 250 paired-end Illumina HiSeq. 1500 platform.

### Assessing the heterozygosity level of the #R1 genome

Illumina reads were trimmed to remove adaptors and filtered by quality using Trimmomatic v0.33^34^. Approximately, 277 million high-quality Illumina reads (Q > 37) (Supplementary Table 1) were used as input to count 21k-mers using Jellyfish v2.3.0^35^, followed by a genome scan using GenomeScope^36^.

### cDNA sequencing

cDNA from flowers of immature #R1 inflorescences was synthesized from 50 ng of total RNA using the SMART-Seq V4 low-input RNA kit (Takara Bio Europe, France). Of the 10 µl reverse transcription reaction, 1 µl was used for quality control and the remaining 9 µl were amplified using Seq Amp DNA Polymerase with seqAmp CB PCR Buffer for long fragment amplification (Takara Bio Europe, France). A sequencing cDNA library with an estimated concentration of 80 fmol (2000 bp average library size) was prepared using the SQK-LSK 109 ligation sequencing kit (Oxford Nanopore Technologies, UK). Preparation included RNA and cDNA purification steps using dAMPure XP Beads (Beckman Coulter, France). RNA quality was assessed using the Agilent High Sensitivity DNA Reagent Kit (Agilent Technologies, France). ONT sequencing and base calling were performed at IRD, as described above. Raw reads were filtered for quality (Q > 10) and length (>300 bp) and trimmed (85 bp at both ends) using Nanofilt v1.0^36^.

### Genome survey and assembly

**Fig. 2:**
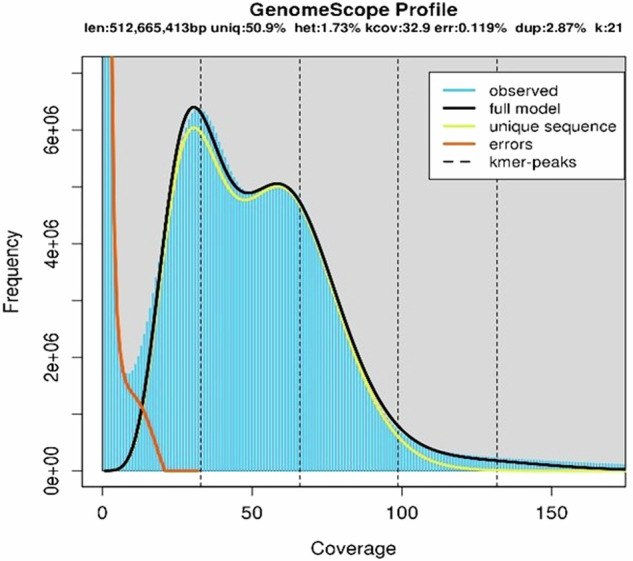
GenomeScope k-mer profile plot of the Illumina #R1 filtered reads. The black line shows the fit of the model to the observed k-mer frequencies (blue graph) at 0-170 coverage scale.

Preliminary k-mer analysis carried out with the Illumina reads predicted a total genome size of 513 Mb, an abundance of repetitive elements of approximately 50.0% and a heterozygosity rate of 1.73%, as indicated by the bimodal k-mer profile (Fig. 2). This high level of heterozygosity was expected for the #R1 genotype based on previous genetic analysis of the natural population from which the plant was collected^29^ and is similar to that reported for other self-incompatible grasses^37,38^. To achieve genome assembly, we first generated 72.13 Gb of ONT long reads (Q > 7) (19.98 Gb from MinION and 52.15 Gb from PromethION) with a N50 = 19.71 kb of read length and a GC content of 45.56% (Supplementary Table 1^39^). The reads were then filtered for quality (Q > 10) and length (>5 kb) using NanoFilt v1.0^33^ resulting in of 68 Gb of data with a %GC of 45.60 and an N50 of 20.41 kb (Supplementary Table 1), which were assembled using Flye v2.9^40^. The *de novo* assembled contigs were polished using Racon v1.4.10^41^ and scaffolded by RagTag v2.1.0^42^ using the available *P. notatum* genome reference^20^ (NCBI Genome assembly ASM2253091v1), excluding the unassigned contigs. The new assembly was polished with the 70 × coverage of Illumina pair-end sequences. Illumina short reads mapping was performed using BWA-MEM v0.7.17^43^, and error correction was performed with Pilon v1.23^44^ in two successive iterations. This procedure resulted in a 557.8 Mb #R1 genome (GenBank GCA_036689595.1), including the ten expected chromosome-length scaffolds (N50 = 56.10 Mb) and a GC content of 45.80% (Table 1). Of the total ONT reads used as input, 99.14% were mapped within the assembly, indicating a high degree of raw data inclusiveness. #R1 pseudomolecules were named based on their sequence similarity to the reference chromosomes^20^. Chromosome size varied between 46.63 and 85.72 Mb, with a mean of 55.78 ± 10.93 Mb (Supplementary Fig. 1). Some of the unassigned contigs reported by Yan *et al*.^20^ showed similarity with sequences within the #R1 chromosomes. These additions probably contribute to the increase in the genome length from 541 Mb of the reference^20^ to 557.8 Mb of the new assembly.

**Table 1:** Summary statistics of *P. notatum* genome assembly and annotation.

#R1 genome assembly parameters	values
Total length (Mb)	557.81
Scaffolds (n)	10
GC content (%)	45.80
N50 scaffolds (Mb)	56.10
Contigs (n)	2,811
N50 contigs (kb)	346.4
Ns (%)	0.05
Heterozygosity (%)	1.73
Repetitive elements (%)	57.86
BUSCO (% total – complete)	98.73 -94.72
BUSCO duplicates (% total – complete)	3.1 -2.9
Predicted gene models (n)	45,074
High confidence gene models (n)	36,079
rRNA (n)	354
tRNA (n)	544
miRNA (n)	59

### Flowers and leaves transcriptome assembly

The #R1 genome was used for a reference-guided transcriptome assembly of flowers and leaves. From a total of 11.9 Gb of ONT cDNA reads from flower transcriptome, ~10 Gb of filtered reads (Q > 10) were assembled using Stringtie v 2.1.4^45^. The resulting flower transcriptome assembly consisted of 36,317 transcripts with a GC content of 51.68% and an N50 of 2,382 bp (Table 2; Supplementary Table 2) (GenBank GKQU01000000.1). Furthermore, the Illumina cDNA paired-end reads (QC > 30) from leaves of diploid genotypes available from NCBI database SRR7347364, SRR7347365, SRR7347366, SRR7347367, SRR7347368, SRR7347369^17^ were reference-based assembled using Trinity v2.0.2^46^ and produced 76,682 transcripts with a %GC content of 46.69% and N50 of 1,545 bp (Table 2, Supplementary Table 2). The features of both transcriptomes were consistent with previous reports for the species^16–18^ and were subsequently used as biological evidence for the #R1 genome annotation (see below).

**Table 2:** Summary of flowers and leaves transcriptome assemblies from diploid *P. notatum* genotypes.

Flower transcriptome assembly of #R1	values
Total length (bp)	70,569,803
No. of transcripts	36,317
GC (%)	51.68
N50 (bp)	2,382
N90 (bp)	1,102
N’s per 100 kb	8.97
**Leaf transcriptome assembly of diploid genotypes**^6^	**values**
Total length (bp)	80,897,606
No. of transcripts	76,682
GC (%)	46.69
N50 (bp)	1,545
N90 (bp)	448
Ns per 100 kb	0

### Genome annotation

#### Repetitive sequences

Repetitive sequences in the #R1 genome assembly were assessed using the filtered Illumina paired-end reads and the RepeatExplorer2 pipeline integrated into the Galaxy platform (https://repeatexplorer-elixir.cerit-sc.cz/) following the protocol described by Novak *et al*.^47^. Briefly, a clustering analysis was performed using RepeatExplorer2 and the TAREAN tandem repeat analyzer module. The DANTE tool was used to extract the consensus sequences of transposable elements (TEs) and classify them based on the REXdb database Viridiplantae 3.0 release^48^, using ‘BLOSUM80’ as scoring matrix and no iterative search. RepeatModeler v4.1.2^49^ (RM2) was used to generate a custom library of *P. notatum* TEs, and RepeatMasker v4.1.2-p1^50^ was used to determine the frequency of repeat DNA families. The RM2 output was then parsed (modified ParseRM.pl script^51^) to identify and quantify TE families. The putative centromeric regions of #R1 chromosomes were localized using the centromere-specific satellite sequences of eight grass species (*Oryza sativa*, *Setaria viridis*, *Setaria italica*, *Panicum hallii*, *Panicum capillare*, *Panicum virgatum*, *Zea mays* and *Zea luxurians*) described by Melters *et al*.^52^. Chromosomal positions were determined by BLASTN analysis^53^ using the satellite sequences as query and considering only the alignments longer than 100 bp and identities >80%^54^. Telomeric regions were identified using the quarTeT tool^55^.

**Fig. 3:**
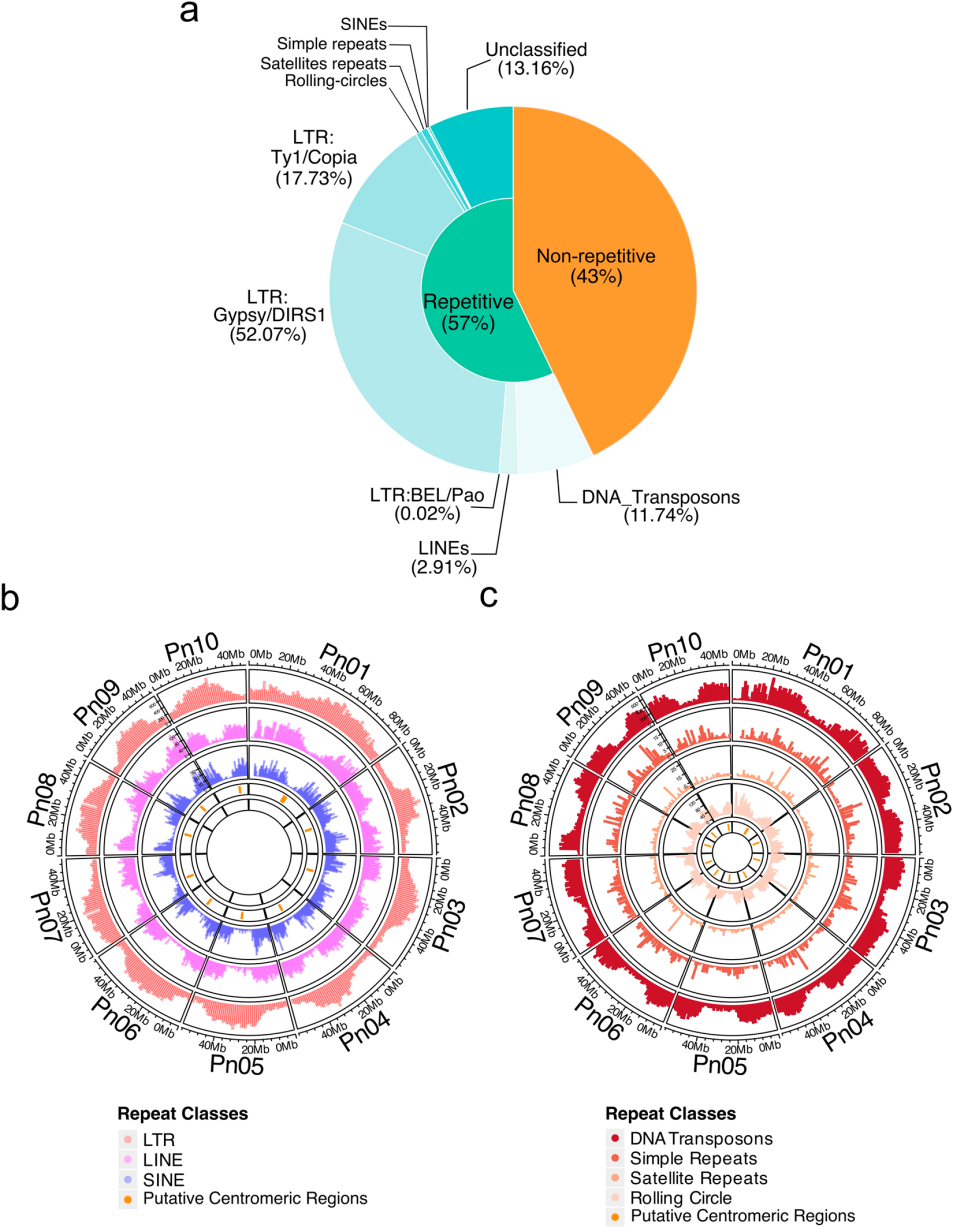
#R1 genomic sequences annotation. (**a**) Percentages of the repetitive and non-repetitive sequences (RepeatMasker results) in the #R1 genome, and proportions of the different types of TEs (estimated over the total of repetitive sequences). (**b,** **c**) Density distribution over the #R1 genome of (**b**) retroelements (from outside to inside LTR, LINE and SINE) and (**c**) DNA transposons, simple repeats, satellite repeats and rolling circles (from outside to inside).

Analysis of the Illumina reads with RepeaExplorer2 identified a total of 320 Mb of repetitive sequences (57.36% of the #R1 assembly), predominantly consisting of retrotransposons (82.12%) and DNA transposons (7.17%) and including a significantly large proportion of unclassified elements (Fig. 3a, Table 3). When mapped onto the #R1 genome, repetitive sequences occupied a minimum of 44.96% (chr. 02) and a maximum of 71.21% (chr. 08) of the chromosome length (Table 4). As expected, the density distribution of the different repeat elements varied along the chromosomes. LTRs were most abundant in putative centromeric regions, whereas retroelements (LINE and SINE), DNA transposons, and rolling circles were prevalent in chromosome arms (Fig. 3b,c). Simple repeats and satellite repeats appeared regularly distributed along all ten chromosomes (Fig. 3c). The putative centromeric regions could be assigned to eight of the ten chromosomes. For chromosomes 2 and 10, these regions could not be properly defined, probably due to a low assembly resolution in these areas and therefore, the proposed locations are hypothetical (Table 4, Supplementary Fig. 1). Similarly, the putative locations of the telomeric regions of chromosomes 2, 3, 4, 9 and 10 were recognized. However, for chromosomes 1, 5, 6, 7 and 8 the positions given are provisional due to the short alignments obtained (Table 4). The average length of the putative telomeres was 6,255 bp, ranging from 70 bp (Chr. 09) to 26,929 bp (Chr. 03) (Table 4).

**Table 3:** Classification of major repeat sequence families in the #R1 genome as assessed using the RepeatMasker software.

Type of Repeat	SubClass1	SubClass2	Number elements	Length occupied	%
Retroelements			168581	234160614	41.98
	SINEs		5547	962897	0.17
	LINEs		23282	9296108	1.66
		R1/LOA/Jockey	618	81749	0.01
		RTE/Bov-B	2374	882133	0.16
		L1/CIN4	20290	8332226	1.49
	LTR elements		139752	223901609	40.14
		BEL/Pao	58	73743	0.01
		Ty1/Copia	44400	56497808	10.13
		Gypsy/DIRS1	93595	166023540	29.76
DNA Transp.			143645	39980268	7.17
	hobo-Activator		17582	4101497	0.74
	Tc1-IS630-Pogo		32360	4909820	0.88
	En-Spm		28307	14342468	2.57
	MuDR-IS905		35612	10783325	1.93
	Tourist/Harbinger		17029	3287179	0.59
	Other		298	21778	0
Rolling-circles			13954	2899921	0.52
Unclassified			161932	41946885	7.52
Total interspersed repeats				316087767	56.67
Satellites			721	3234032	0.58
Simple repeats			1284	529555	0.09

Total masked bases: 322,751,275 bp (57.86%). Percentages of repetitive sequences were estimated from the occupied length of each type of repetition over the total length of the assembly.

**Table 4:** Length and proportion of repetitive elements of the *P. notatum* #R1 chromosomes.

Chrom.	Length (Mb)	No. of Contigs	Repetitive elements (%)	Putative Centromeric location (bp)	Putative telomeric location up (bp)	Putative telomeric location down (bp)
Chr01	85.72	514	70.44	53,925,281-54,491,081	0–105	85,722,103-85,722,362
Chr02	57.20	219	44.96	23,848,602-23,848,862	0–23,380	57,200,198-57,200,359
Chr03	58.66	287	55.57	23,440,597-23,520,834	0–26,929	58,652,041-58,660,280
Chr04	56.06	281	53.44	33,230,447-33,265,194	0–5,852	56,058,201-56,063,745
Chr05	56.49	252	61.52	32,764,408-32,961,888	0–196	56,488,357-56,488,469
Chr06	52.89	284	64.82	27,241,750-27,814,554	0–161	52,880,625-52,892,378
Chr07	50.47	243	60.02	19,942,834-20,988,206	0–182	50,474,816-50,474,921
Chr08	48.77	232	71.21	27,232,006-30,535,342	0–98	48,768,635-48,768,754
Chr09	44.90	253	61.54	20,860,291-21,218,959	0–17,073	44,903,870-44,903,940
Chr10	46.63	246	61.66	35,353,092-35,353,223	0-1,743	46,613,857-46,636,880

#### Gene annotation

Gene prediction and annotation were performed using the MAKER v2.31.9 pipeline^56^ by integrating *ab initio* gene model predictions with biological transcriptomic and proteomic data through multiple BLAST steps using Exonerate v2.4.0^57^. The soft-repeat-masked version of the #R1 genome together with flower and leaf transcriptomes (this work) merged and filtered for redundancy (similarity threshold of 90%) using CD-HIT^58^ were used as input. In addition, the transcriptome of *Sorghum bicolor* NCBIv3 (GeneBank GCA_000003195.3) and the proteome of *Oryza sativa* Japonica Group cv. Nipponbare (Genebank GCA_001433935.1) were included as expressed sequence evidence of related species. Two MAKER iterations were performed to obtain the final annotation. In the first one, *ab initio* gene predictions were carried out using AUGUSTUS v3.2.2^59^ with the EST trust-blindly option enabled and *Oryza sativa* as the model species. The resulting gene models were filtered to retain only those with an annotation error distance (AED) <0.5^56^. The outcome of this first annotation was then used to train new species models for AUGUSTUS and SNAP^60^ for the second run of MAKER. Gene models with an AED score > 0.5 and transcripts <50 nt were filtered out. The predicted coding sequences (CDS) obtained with MAKER were then translated to protein sequences using the program GffReadv0.12.7^61^ with parameter “-y”. Predicted protein sequences were checked for CDS features (presence of start and stop codons) and for homology with known domains using InterProScan v5.53.87.0^62^ (consulting the databases TIGRFAM, SFLD, SUPERFAMILY, PANTHER, SMART, CDD, PIRSR, Pfam, on April 2023). Gene models that fitted with both criteria were considered as “high confidence”.

**Fig. 4:**
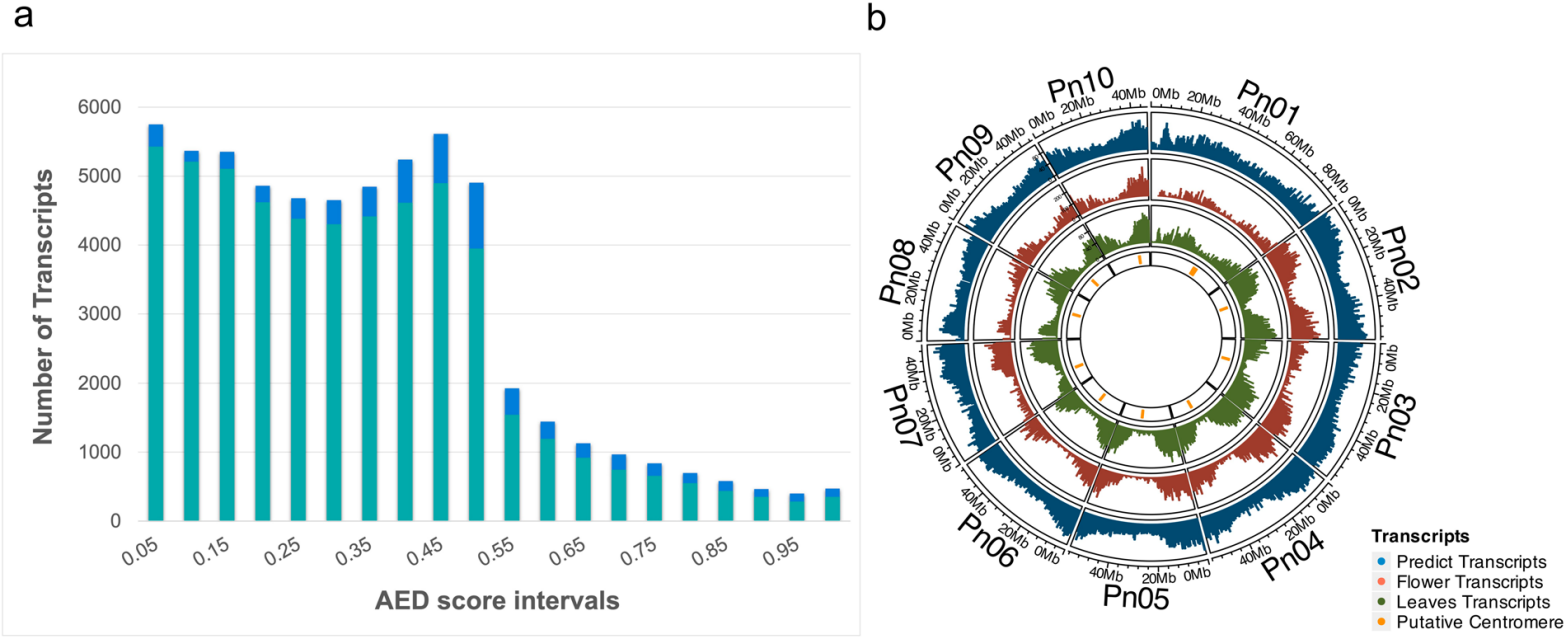
Transcripts prediction and distribution: (**a**) Histogram of the Annotation Edit Distances (AED) scores of the predicted transcripts. Green and blue bars show the number of transcripts with and without hits in the InterProScan database, respectively. (**b**) Circle plot showing (from outside to inside) the density distribution of predicted, leaf and flower transcripts in the #R1 genome.

Using this strategy, a total of 51,249 transcripts with an AED < 0.5 (85.18% of the total predicted) (Fig. 4a), which defined 45,074 gene models with approximately 1.14 transcripts per gene, were obtained (Supplementary Table 3). The average lengths of mRNA and CDS were 3,679 nt and 1,258 nt, respectively. Each predicted gene contained an average of 4.4 exons, and the exons’ mean length was 346 nt. Of the total predicted gene models, 36,079 (80.04%) were classified as high-confidence (HC) genes. The complete list of genes, their genomic coordinates and corresponding *A. thaliana* and rice homologs, together with their functional annotation, are summarized from the GFF file in the Supplementary Table 3. As expected, over 99% of the flower and leaf transcripts mapped in the #R1 genome showing a high density towards the ends of the chromosome arms and a low density in most of the putative centromeric regions (Fig. 4b). The number and density of genes per #R1 chromosome are shown in Table 5.

**Table 5:** Number and density of predicted genes per #R1 chromosomes.

Chromosome	Gene number	Genes per Mb
Chr01	5,742	66.99
Chr02	5,538	96.82
Chr03	5,027	85.70
Chr04	5,048	90.05
Chr05	4,687	82.97
Chr06	4,171	78.86
Chr07	4,173	82.68
Chr08	3,335	68.38
Chr09	3,636	80.98
Chr10	3,717	79.71

#### Identification of rRNA and tRNAs

rRNA genes were identified using Barrnap v0.9^63^ software with an e-value cut-off for similarity of 1e^−10^ and a minimum length threshold of 0.9. In addition, tRNA genes were identified using tRNAscan-SE V1.3.1^64^ with the ‘-infernal’ mode. These analyses resulted in the annotation of 354 rRNA genes and 544 tRNA genes in the #R1 genome (Table 1), which localization is presented in the GFF annotation file deposited in the NCBI database accession number (GCA_036689595.1).

#### Prediction of microRNA (miRNA) genes and targets

MicroRNA (miRNA) gene precursors present in the #R1 genome were searched using the small RNA (sRNA) sequence database of the reproductive development of sexual and apomictic *P. notatum* genotypes^19^ available at the NCBI BioProject Accession: PRJNA373857 and the software ShortStack 3.8.4^65^. miRNA precursors, miRNA mature sequences and putative targets in the #R1 genome were detected as described in Ortiz *et al*.^19^ using the #R1 assembly as a reference. The putative miRNA’s target regions were analyzed using the #R1 GFF annotation file to determine the location of the mature miRNA alignment (5′ UTR, exon, intron, or 3′ UTR regions) within the genes. Following these procedures, a total of 59 clusters distributed across the 10 chromosomes containing sRNAs were detected (Supplementary Table 4, sheet 1), most of them producing mature miRNAs of 21 nt (47 clusters) and 22 nt (9 clusters). A total of 52 unique mature miRNAs were predicted, corresponding to 21 known families and including all miRNAs previously described in the species, with the exception of the miR390^19^ (Supplementary Table 4, sheet 2). Moreover, two new miRNAs (miR827 and miR3979) were identified in the species (Supplementary Table 4, sheet 2). Fourteen precursors generate putative mature miRNAs with no significant match in MirBase and, therefore, may represent novel *Paspalum*-specific miRNAs. A search for target regions in the #R1 genome performed with TargetFinder^66^ identified 1,456 unique genomic regions (TF score < 4), of which 1,324 have homology with known proteins (Supplementary Table 4, sheet 3).

## Data Records

The raw reads derived from the #R1 genome sequencing using Oxford Nanopore (ONT) technology were deposited in the NCBI Sequence Read Archive (SRA) database under accession Nos. SRS19975480^67^ and SRS19975482^68^. The sequencing Illumina raw data were deposited in the NCBI SRA database SRS19975483^69^ and SRS19975484^70^. The #R1 genome assembly and annotation were deposited in the NCBI database under accession No GCA_036689595.1^71^. The reads of the #R1 flower cDNA ONT sequencing were deposited in SRA database SRS19975481^72^, and the #R1 flower transcriptome assembly were deposited in the NCBI database under accession No. GKQU00000000.1^73^. The raw reads from leaves were downloaded from the NCBI Sequence Read Archive (SRA) database accession Nos. SRR7347364^74^, SRR7347365^75^, SRR7347366^76^, SRR7347367^77^, SRR7347368^78^, SRR7347369^79^. The leaf transcriptome assembly was deposited in the NCBI under the accession number DAWXED000000000^80^. The precursor and mature miRNA sequence data recovered from the #R1 genome has been incorporated in the Supplementary Table 4, sheets 1 and 2.

## Technical Validation

### Assessing the quality of HMW genomic DNA for ONT sequencing

The quality and integrity of the #R1 genomic DNA for ONT sequencing was evaluated using a NanoDrop One/One Spectrophotometer and a Pulsed-Field Gel Electrophoresis system (PFGE BioRad) according to Mariac *et al*.^30^ (https://www.protocols.io/view/high-molecular-weight-dna-extraction-from-plant-nu-83shyne). DNA preparations consistently showed spectrophotometric ratios 260/280 nm 1.8–2.0 and 260/230 2.0-2.2, confirming the purity of the extraction. On the other hand, the high molecular weight of the DNA preparation was checked out by loading 1.5–5.5 µg of genomic DNA in 1% agarose gel (0.5 × TAE) with 5 µl of 6 × loading buffer and electrophoresed using the following parameters: pulse time: initial = 5 s, final = 117 s, running time = 20.5 h, V/cm = 5, Angle = 120, Temp = 14° and mA end of run = 255. The molecular weight of the genomic DNA preparation obtained ranged from 48 to 200 kb (Supplementary Fig. 2).

### Assessment of genome and transcriptome assembly and annotation quality

The NCBI-FCS-GX scan tool^81^ was used to find contaminants in the assembly, setting the taxon in *Viridiplantae*. In addition, the presence of organellar DNA was assessed by BLASTn analysis (query coverage >30% and % of identity >60%) using the *Oryza sativa* IRGSP-1.0 organellar data set as query. No contaminants or organellar DNA were detected in the #R1 assembly. The software Merqury^82^ was used to estimate the base-level accuracy and k-mer completeness of the #R1 genome. This analysis showed an assembly consensus quality value (QV) of 30.2, which correspond to an accuracy of 99.9%, and a k-mer completeness value of 84.3%. Nevertheless, we cannot discard that some regions may include both haplotypes (Supplementary Fig. 3). In addition, the #R1 assembly quality was evaluated using BUSCO v5^83^ using the Liliopsida gene set as a reference, and by mapping the Illumina paired-end reads over the genome. The BUSCO score showed the presence of 94.7% of complete genes,(with 91.8% of them corresponding to single genes), 4% of fragmented genes and 1.3% of missing genes (Supplementary Fig. 4a). Furthermore, the percentage of the total core genes with more than one ortholog was only 3.1%. Moreover, 97.7% of the paired-end Illumina reads were properly mapped by BWA-mem v0.7.17 to the #R1 genome, with an estimated average coverage depth of 93.2×. Using the same procedure for assessing MAKER gene annotation, the BUSCO score showed that 94.4% of the 3,236 Lilliopsida single-copy genes were properly annotated, with an average of 1.19 orthologs for each gene (Supplementary Fig. 4b). In this case, the percentage of duplicate transcripts increase up to 10.1%, probably due to the inclusion of splicing variants. On the other hand, BUSCO analysis performed to evaluate both the flower and leaf transcriptome assemblies revealed 87.9% and 82.2% of complete, 3.7% and 8.6% of fragmented, and 8.4% and 9.2% of missing genes, respectively (Supplementary Fig. 4c,d). Overall, these results indicate that both transcriptomes have a high level of completeness, and therefore represent comprehensive evidence of the expressed sequences of the #R1 genome.

**Table 6:** Software and parameters used during the #R1 genome sequencing, assembly and annotation.

Software	Parameters	Reference
guppyGPU v6.0.6	-c dna_r9.4.1_450bps_hac.cfg -r --num_callers 4 --gpu_runners_per_device 8 --qscore_filtering --min_qscore 7 -x cuda:3	https://github.com/nanoporetech
NanoPlot v1.31.0	default	^33^
Flye v2.9	--threads 32 --nano-hq --genome-size 600 m	^40^
Racon-gpu v1.4.10	default	^41^
RagTag v2.1.0	scaffold -C -r --aligner minimap2 --mm2-params -x asm5	^42^
Bwa v0.7.17	mem	^43^
Pilon v1.23	--fix all --changes --diploid	^44^
Jellyfish v2.3.0	count -C -m 21 -s 10000000000 -t 12 | histo -h 10000000 -t 10	^35^
GenomeScope	kmer = 21, read length = 200, max kmer coverage = 50.000.000	^36^
BUSCO v5	ortholog set = OrthoDB v10 – Liliopsida	^83^
Trimmomatic v0.33	PE -phred33 CROP:230 ILLUMINACLIP:Illumina SLIDINGWINDOW:4:30	^34^
RepeatExplorer2 v4.1.2	default	^47^
RepeatModeler v4.1.2	-database R1_rep -engine rmblast	^49^
RepeatMasker v4.1.2	-e rmblast -nolow -norna -pa 4 -s -html -gff -lib R1_rep-families.fa	^50^
stringtie v2.2.1	-L–ref	^45^
Trinity v2.0.2	--seqType fq --max_memory 30 G --CPU 4 --min_contig_length 300 --group_pairs_distance 500 --no_version_check --verbose --full_cleanup --genome_guided_bam --genome_guided_max_intron 5000	^46^
NanoFilt v1.0	-q 10 -l 5000 (DNA) -q 10 -l 300 --headcrop 85 --tailcrop 85 (cDNA)	^33^
BLAST + /2.13.0	-evalue 0.00001 -perc_identity 80 -qcov_hsp_perc 80 (centromeric regions)	^53^
quarTeT v1.2.1	TeloExplorer -c plant -m 5	^55^
MAKER v2.31.9	max_dna_len = 100000 min_contig = 1 pred_flank = 200 alt_splice = 1 split_hit = 10000 single_exon = 1 single_length = 250 est2genome = 1 (run 1) est2genome = 0 (run 2)	^56^
Exonerate v2.4.0	integrated to MAKER pipeline	^57^
CD-HIT v4.8.1	Cd-hit-est -c 0.9 -n 8 -d 0 -T 8 -M 1000	^58^
AUGUSTUS v3.2.2	integrated to MAKER pipeline: augustus_species = rice (run 1) augustus_species = custom (run2)	^59^
SNAP	integrated to MAKER pipeline: snap_hmmm = custom (run 2)	^60^
GffReadv0.12.7	-y -x / -J (high confidence genes)	^61^
InterProScan v5.53.87.0	-appl TIGRFAM,SFLD,SUPERFAMILY,PANTHER,SMART,CDD,PIRSR,Pfam,MobiDBLite -f TSV,GFF3 -goterms	^62^
Barnap v0.9	--reject 0.9 --lencutoff 0.9 --evalue 1e-10 --kingdom eukA9:AMJ9	^63^
tRNAscan-SE v1.3.1	--infernal	^64^
ShortStack v3.8.4	--mismatches 2 --bowtie_cores 6	^65^
TargetFinder	-r -t 20 -c 4	^66^
NCBI-FCS-GX	--tax-id “1”--div “plnt:plants”--split-fasta “true”--gx-db gxdb --action-report true	^81^
Merqury	default / meryl kmer = 21	^82^

### Supplementary information

Supplementary Table 1

Supplementary Table 2

Supplementary Table 3

Supplementary Table 4

Supplementary Figures 1_4

## Data Availability

All software packages used in this study were run according to their user manuals. The version and parameters used are listed in the Table 6. No specific custom codes were used in this study.
