# Supplementary material for: Chromosome-scale genome assembly and annotation of Paspalum notatum Flüggé var. saurae
Source: Sci Data. 2024 Aug 16;11:891. doi: 10.1038/s41597-024-03731-0 (PMC11329641; doi:10.1038/s41597-024-03731-0)

Supplementary Figures

Supplementary Figure 1: Diagrams of the 10 chromosomes of *P. notatum* showing the length and the putative positions of the centromeric and telomeric regions.

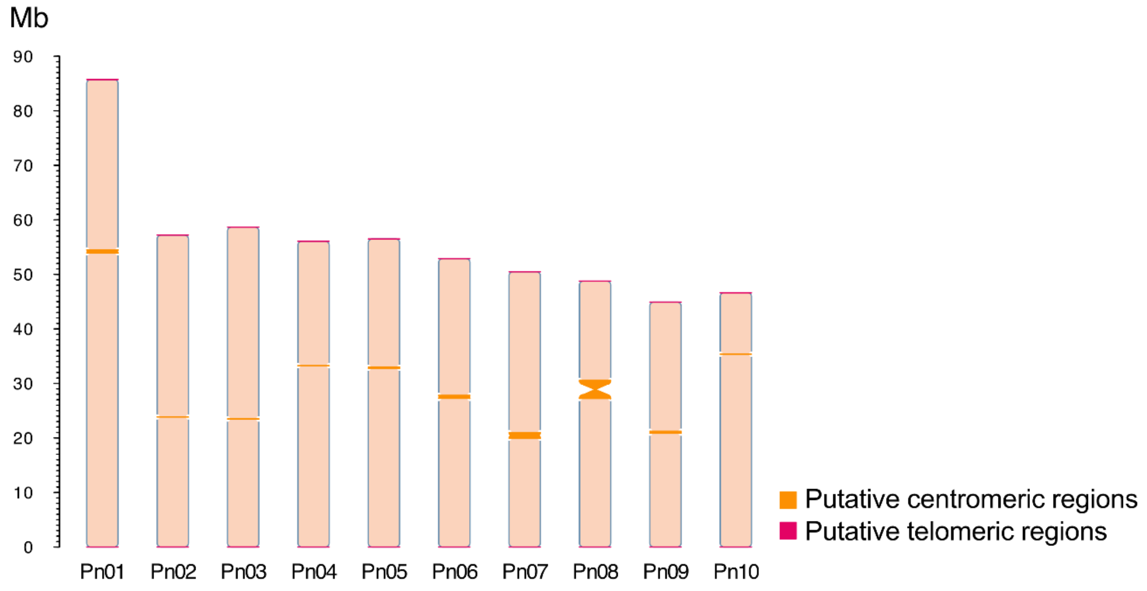

Supplementary Figure 2: PFGE of genomic DNA of *P. notatum* #R1 for ONT sequencing.

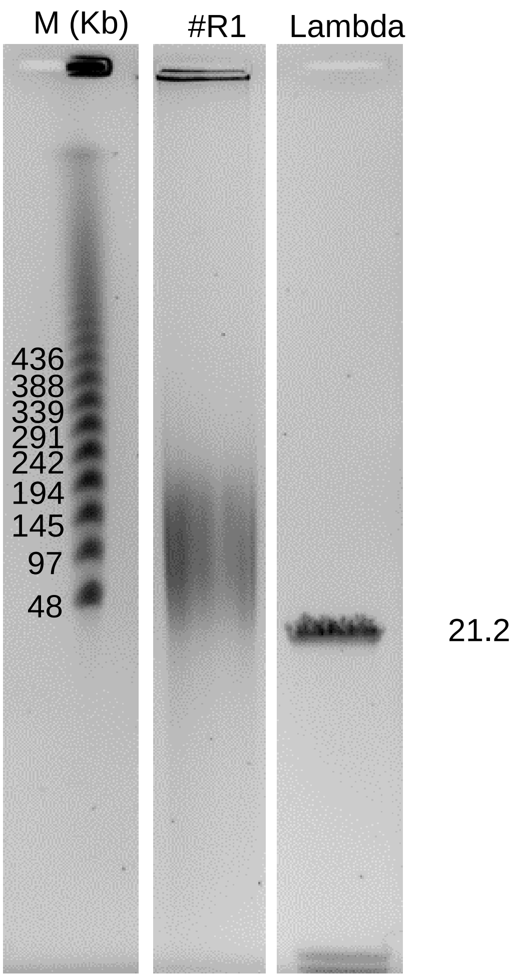

M= Lambda PFG Ladder (New England Biolabs, Inc.)

Supplementary Figure 3: Assembly spectra plot obtained with the Merqury software[74]. The Illumina reads were used to make a k-mer database using Meryl and then along with the #R1 assembly used as input for Merqury.

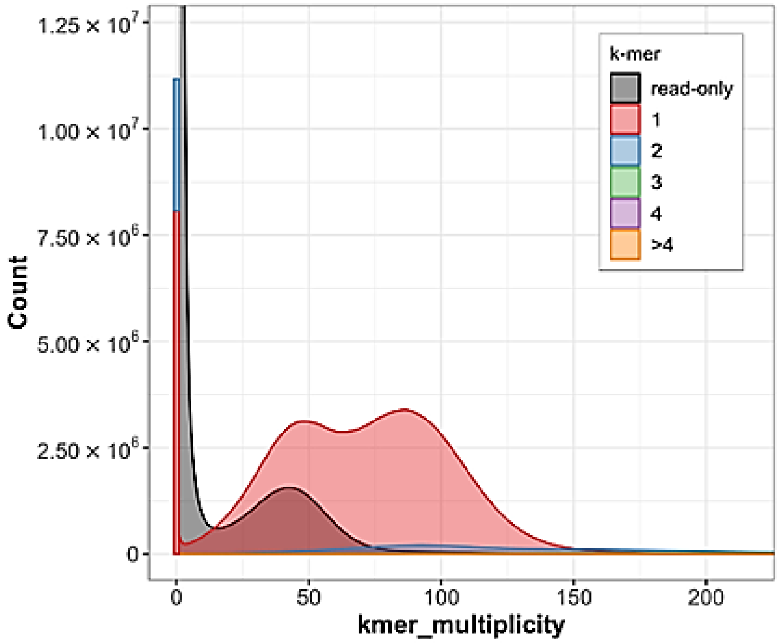

Supplementary Figure 4: BUSCO score of the #R1 *Paspalum notatum* genome and transcriptome assemblies. a) #R1 long reads ONT genome assembly, b) MAKER predicted transcriptome, c) transcriptome of inflorescences assembled in this work, and c) transcriptome of leaf tissue assembled in this work.

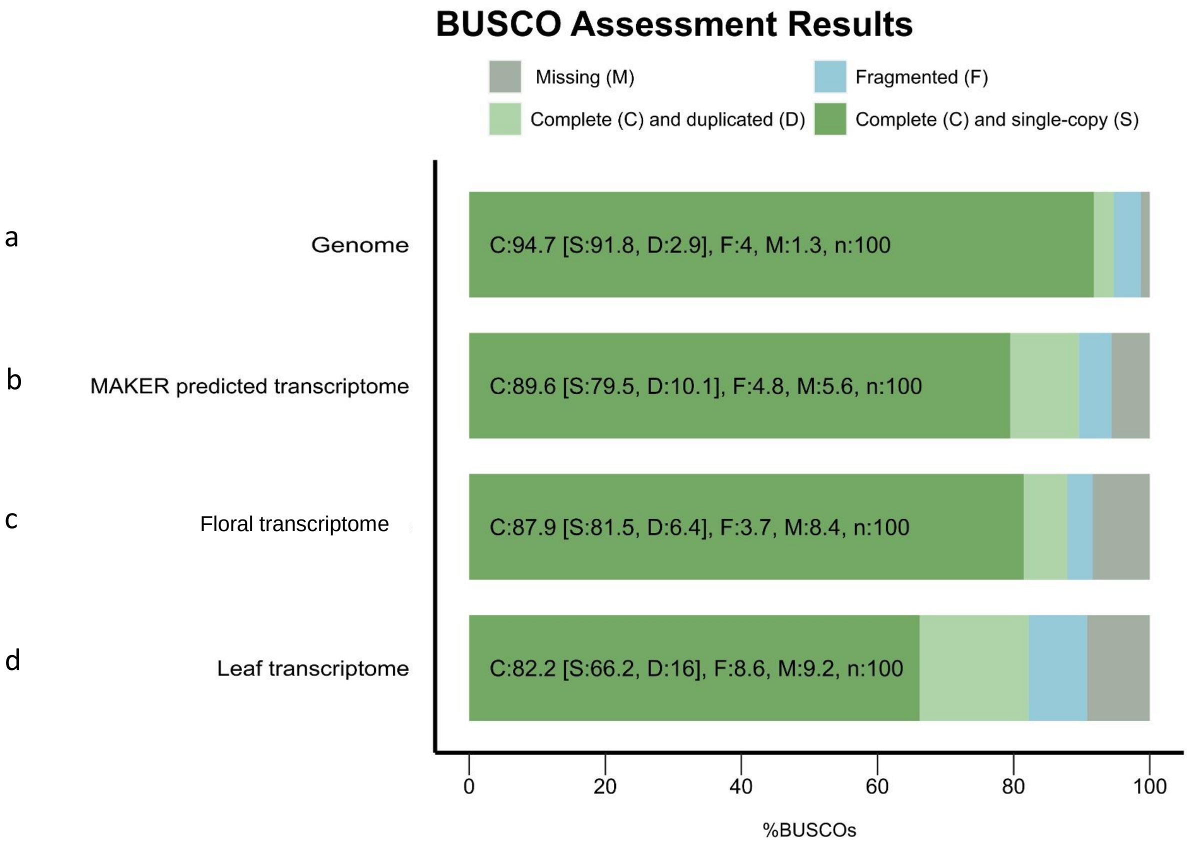

Supplement: Supplementary file 5 — Supplementary Figures 1_4 [file 41597_2024_3731_MOESM5_ESM.pdf]
